# Impella-Supported Off-Pump Coronary Artery Bypass Grafting (CABG) in a Patient With Severe Mitral Regurgitation: A Case Report

**DOI:** 10.7759/cureus.85315

**Published:** 2025-06-04

**Authors:** Keisuke Sumii, Hiroe Hamaguchi, Koichiro Goto

**Affiliations:** 1 Anesthesiology, Saitama Sekishinkai Hospital, Sayama, JPN

**Keywords:** extracorporeal membrane oxygenation, impella, intra-aortic balloon pumping, mitral regurgitation, off-pump coronary artery bypass grafting

## Abstract

Perioperative cardiogenic shock can be fatal, and assisted circulation with an Impella percutaneous ventricular support pump catheter (Abiomed, Danvers, MA, USA) is useful in patients with severely compromised cardiac function. However, we report a case in which an Impella implanted preoperatively deepened its position during off-pump coronary artery bypass grafting decannulation. This change in position caused the Impella to interfere with the posterior mitral valve leaflet and led to severe mitral regurgitation and difficulty with intraoperative circulatory control. In patients without significant left ventricular enlargement, the heart team should discuss the risk of interference with the mitral valve and inhalation failure during decannulation. In such cases, other forms of assisted circulation, such as intra-aortic balloon pumping or cardiopulmonary bypass, should be considered.

## Introduction

Perioperative cardiogenic shock has an extremely poor prognosis in patients undergoing cardiac surgery. Single-use intra-aortic balloon pumping (IABP) and its combination with extracorporeal membrane oxygenation (ECMO) are often performed for perioperative assisted circulation in patients with moderate or higher cardiogenic shock; however, the efficacy is limited in patients with major cardiac dysfunction [1,2]. In contrast, a percutaneous ventricular support pump catheter (Impella®; Abiomed, Danvers, MA, USA) showed good treatment outcomes for patients with perioperative cardiogenic shock by reducing left ventricular (LV) afterload and increasing systemic organ perfusion, leading to the use of Impella-supported coronary artery bypass grafting (CABG) [3,4].

The operative mortality of 0.7% with off-pump CABG (OPCAB) is low, but the 4% mortality in converting to cardiopulmonary bypass is higher [5]. The appropriate choice of the circulation assistance method and operative procedures for each patient requires careful discussion among heart teams, including cardiovascular surgeons and cardiologists. Although complications of mechanical mitral valve injury associated with Impella have been reported in the past, to the best of our knowledge, this is the first report of such an event occurring due to positional displacement associated with heart displacement during OPCAB [6].

We experienced a patient who had good preoperative Impella-assisted circulation, but who developed severe mitral regurgitation (MR) due to a narrowing of the LV cavity following heart displacement of the OPCAB, which caused the posterior wall of the left ventricle to approach the tip of the Impella inlet and interfere with the tendon cords at the posterior leaflet of the mitral valve, causing the valve to become open and fixed.

## Case presentation

**Figure 1:**
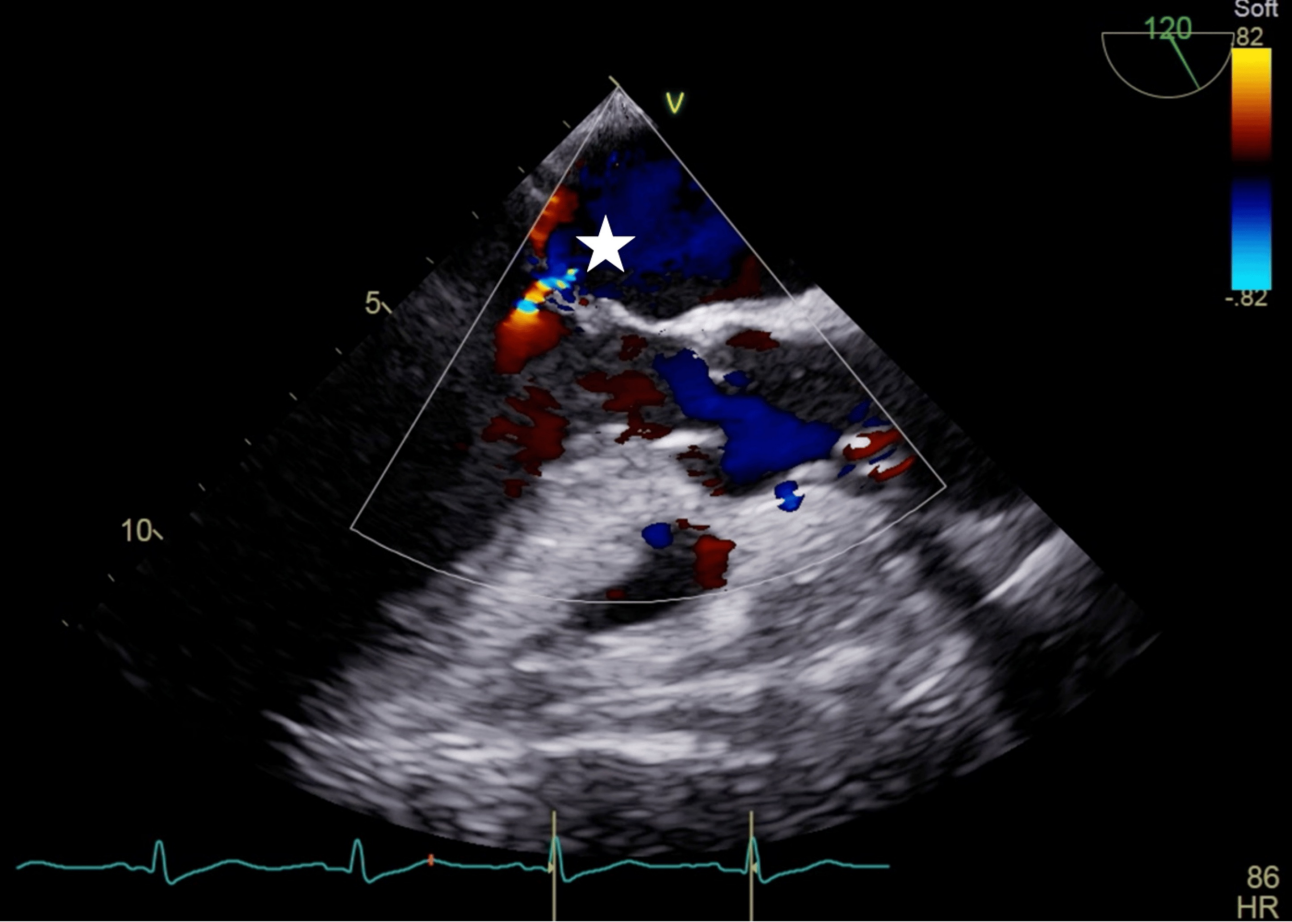
TEE findings after anesthetic induction. The LVEF was 40%, and there was a mild hypokinesis of the left ventricular motion and mild mitral regurgitation flow centrally from the commissure of the mitral valve (asterisk). TEE: transesophageal echocardiography; LVEF: left ventricular ejection fraction

A male patient in his 70s (height: 158 cm, body weight: 57 kg) with a past medical history of hypertension, dyslipidemia, chronic kidney disease, and ischemic heart disease was transported to our hospital by emergency ambulance due to chest pain and dyspnea that had developed on the same day. Preoperative transthoracic echocardiography (TTE) showed a left ventricular ejection fraction (LVEF) of 40% and severe wall motion loss in the lateral and inferior walls of the left ventricle with no catecholamine support. Chest X-ray showed a cardiothoracic ratio of 46%, with no pulmonary congestion or pleural effusion. Coronary angiography (CAG) indicated three-vessel disease, which includes left main trunk stenosis (99%), left circumflex artery stenosis (99%), and right coronary artery root stenosis (99%). Thus, an Impella Cardiac Power (CP) device was inserted from the right femoral artery under fluoroscopic guidance. The “big catheter” of the Impella was confirmed to face the direction of the apex and provided assisted circulation of 3.3-3.5 L/min. We decided to perform emergency OPCAB using the indwelling catheter. Transesophageal echocardiography (TEE) after anesthesia induction showed LV circumferential hypokinesis and a slight mitral regurgitation (MR) central jet from the mitral valve commissure, with LVEF 40%, but there were no valvular degenerative changes in the mitral valve (Figure 1). The suction tip of the Impella was 3.7 cm from the aortic annulus. The LV internal end-diastolic diameter (LVIDd) was 25 mm, and the LV internal end-systolic diameter (LVIDs) was 18 mm.

**Figure 2:**
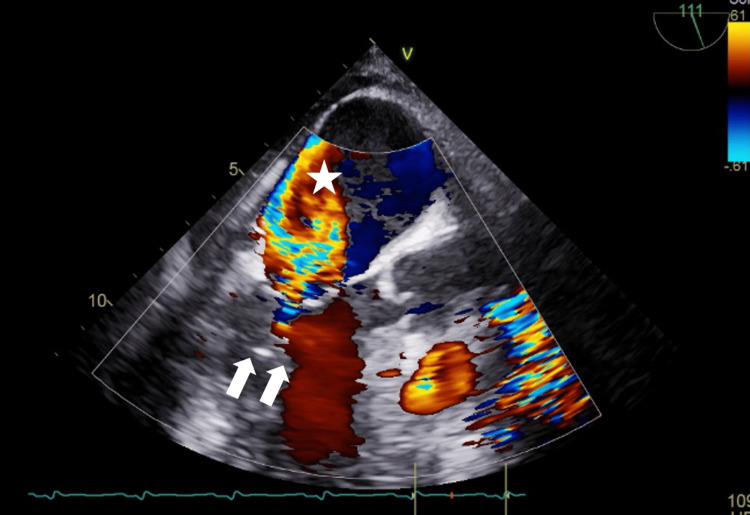
TEE findings after intraoperative heart displacement. Due to the narrowing of the left ventricular cavity caused by heart displacement, the tip of the Impella inlet (arrow) obstructed the supporting tissue of the posterior apex of the mitral valve, resulting in open fixation of the mitral valve and poor junction with the anterior apex, leading to severe MR (asterisk). TEE: transesophageal echocardiography; MR: mitral regurgitation

During surgery, the Impella inlet approached the posterior wall of the left ventricle due to the narrowing of the LV cavity caused by heart displacement, resulting in interference with supporting tissues of the posterior mitral leaflet. Consequently, the patient developed severe MR associated with eccentric jets from the commissure region of the mitral valve toward the left atrial posterior wall (Figure 2). Although the patient's hemodynamics were stable after induction, 90 minutes later, they collapsed due to heart displacement. Systolic blood pressure (SBP) decreased from 99 mmHg to 41 mmHg with heart displacement, and regional oxygen saturation (rSO2) in the right and left cerebral arteries decreased from 81% to 60% and from 75% to 55%, and partial pressure of arterial oxygen (PaO2) in the right upper extremity decreased from 394 mmHg to 160 mmHg. Approximately three minutes later, central veno-arterial extracorporeal membrane oxygenation (V-A ECMO) was started at 3.0 L/min.

**Figure 3:**
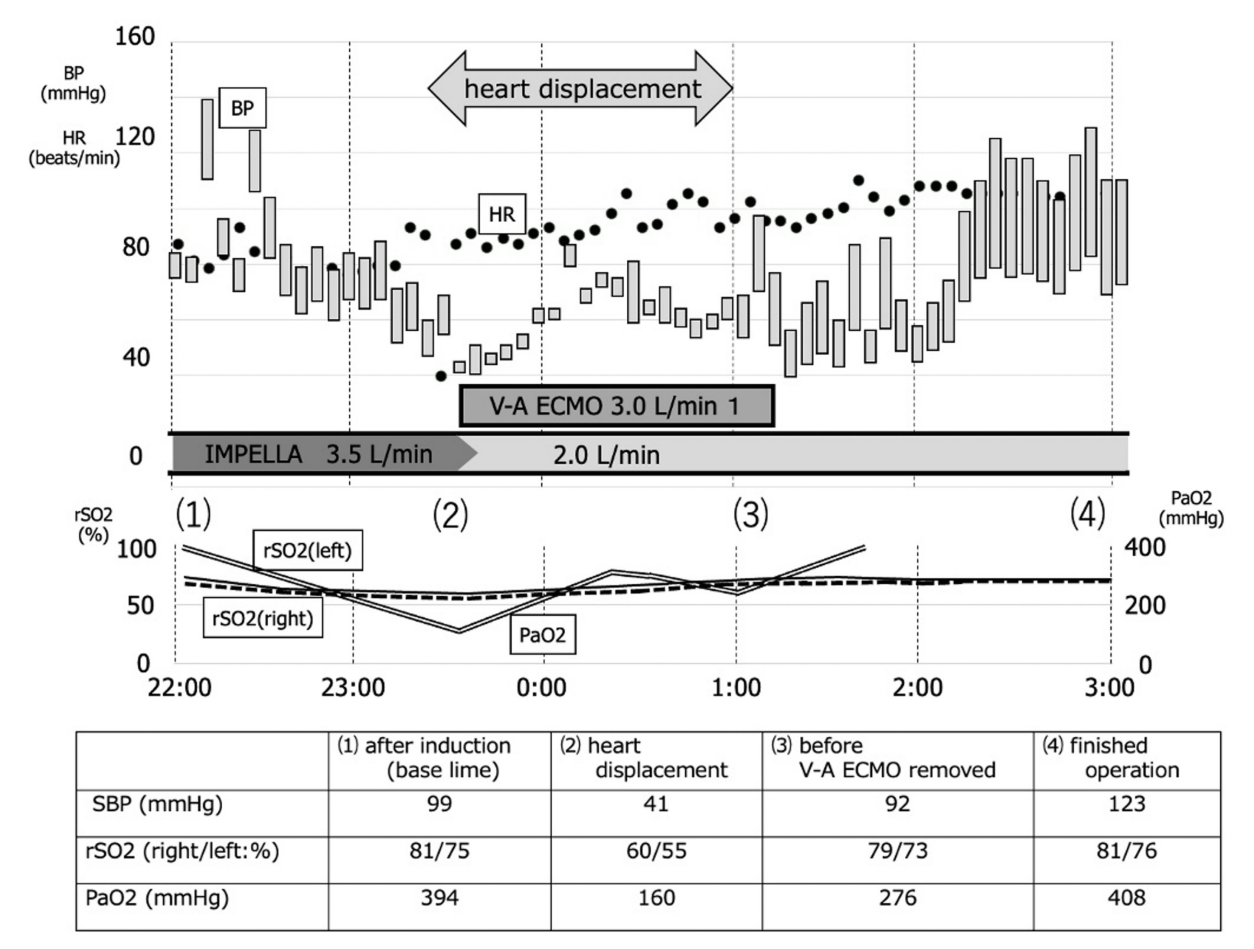
Changes in blood pressure (BP), heart rate (HR), rSO2 and PaO2 during surgery. The plots show BP (mmHg, left longitudinal axis), HR (times/min, left longitudinal axis), rSO2 (%, left longitudinal axis; left: solid line, right: dotted line), and PaO2 (mmHg, right longitudinal axis; double line). Severe mitral regurgitation developed due to the Impella inserted before surgery interfering with the posterior mitral leaflet after manipulation for cardiac conversion, resulting in a sudden drop in BP. After initiation of ECMO, BP increased, and then bilateral rSO2 and PaO2 in the right upper also increased. After anastomoses, BP and PaO2 were confirmed to be stable, and there was improved cardiac contraction. Consequently, ECMO was removed, and the surgery was completed. SBP, rSO2, and PaO2 at (1) after induction of anesthesia, (2) heart displacement, (3) before V-A ECMO removal, and (4) finished operation, marked in the graph above, are shown in the table, respectively. rSO2: regional oxygen saturation; PaO2: partial pressure of arterial oxygen; ECMO: extracorporeal membrane oxygenation; SBP: systolic blood pressure; V-A ECMO: veno-arterial extracorporeal membrane oxygenation

**Figure 4:**
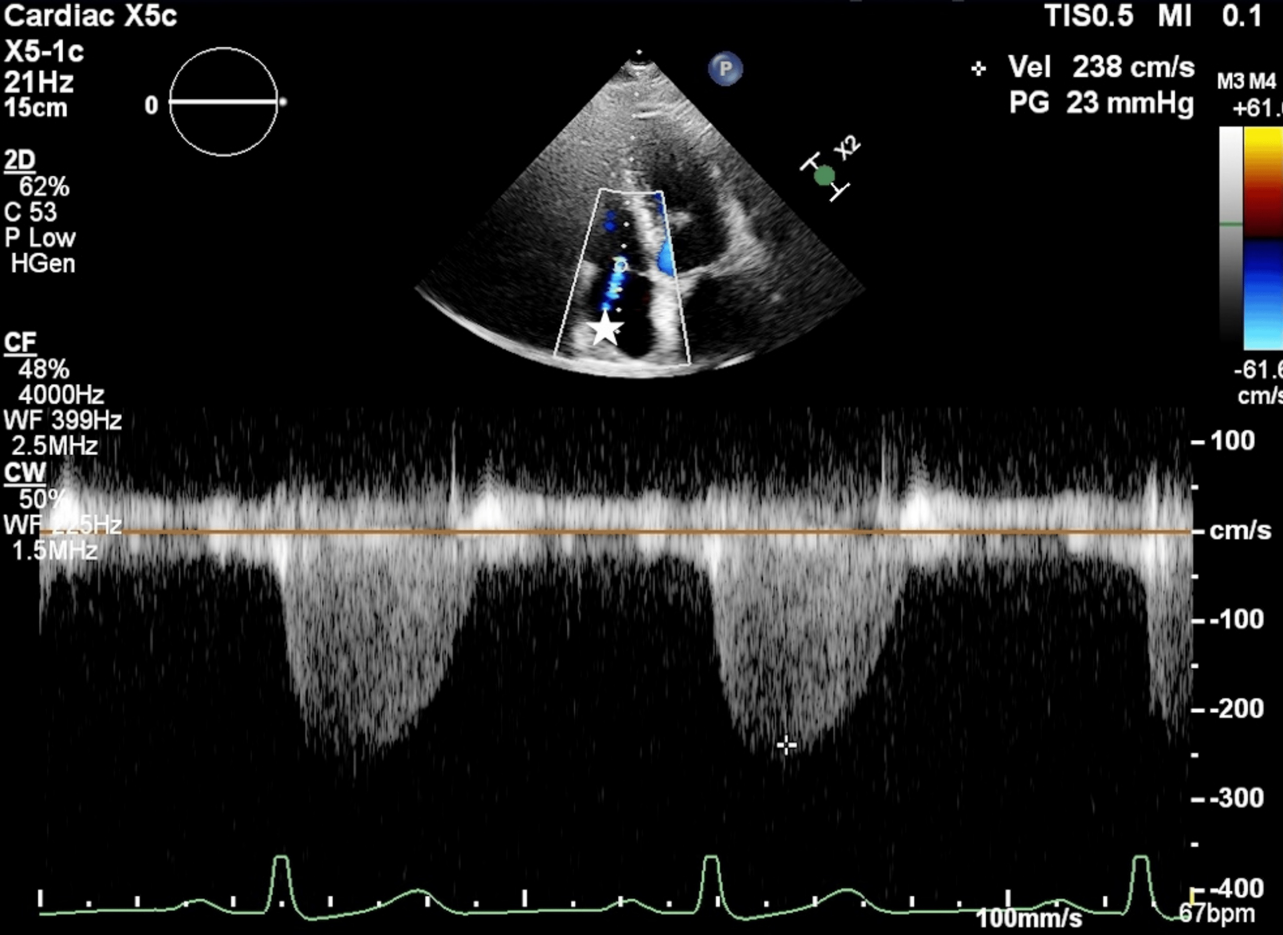
TTE follow-up findings on postoperative day 8. TTE revealed a LVEF of 52% and severe wall motion loss in the lateral and inferior walls of the left ventricle with no catecholamine support. A mild MR jet was observed centrally from the commissure (asterisk). The maximum flow velocity of MR was 2.38 m/s, and the mean pressure gradient was 23 mmHg. There was no impairment of the supporting tissues of the valve, such as tendon cords or papillary muscles. TTE: transthoracic echocardiography; LVEF: left ventricular ejection fraction; MR: mitral regurgitation

After initiation of ECMO, perfusion pressure was stabilized, and subsequently, the Impella volume was reduced to 2.0 L/min in order to prevent further MR exacerbation and damage due to LV aspiration by Impella, and the target perfusion volume was achieved by the sum of ECMO and Impella flow rates. Bilateral intracranial rSO2 and PaO2 in the right upper extremity recovered, and no change in ECG or deteriorated movement disorder in the LV wall was found. It was considered that organ perfusion was not a problem, and cardiac conversion was resumed (Figure 3). First, a central anastomosis of the ascending aorta and the great saphenous vein graft was performed; then, an anastomosis of the right internal thoracic artery to the anterior descending branch of the left coronary artery was performed by axis torsion. Further, an anastomosis of the left internal thoracic artery to the obtuse marginal branch of the left coronary artery was performed by twisting the axis, and an anastomosis of the great saphenous vein graft to the posterior descending branch of the left coronary artery was performed by heart inversion. After 60 minutes from ECMO introduction, all anastomoses were performed, and full hemostasis was confirmed. Lactate levels increased to 2.82 mmoL/L during heart displacement, but decreased to 2.12 mmoL/L when hemostasis was confirmed. SBP increased to 90 mmHg, and PaO2 increased to 408 cmH2O under ECMO off. Hemodynamics were stable, and ECMO was carefully removed. The operative time was 4 hours 23 minutes. Intraoperatively, the circulation was maintained with about 0.06 γ of noradrenaline and 1 γ of dobutamine. Postoperatively, TTE showed no impairment of the mitral tendon cords or papillary muscles requiring therapeutic intervention, and the Impella returned to its correct position and improved to mild MR. Slight positioning of the Impella CP was difficult and was not performed due to the risk of dislocation and hemodynamic compromise. The patient was weaned from Impella on postoperative day (POD) 4, extubated from mechanical ventilation on POD 7. POD 8, TTE showed a LVEF of 52% and severe wall motion loss in the lateral and inferior walls of the left ventricle with no catecholamine support. A mild MR jet was observed centrally from the commissure, with no impairment of the supporting tissues of the valve, such as the tendon cords or papillary muscles (Figure 4). And the patient was discharged from the intensive care unit (ICU) on POD 13.

## Discussion

Efficacy has been shown for Impella for cardiogenic shock after cardiac surgery and Impella-supported CABG during surgery [7-10]. However, patients with reduced LV dilatation, even with good assisted circulation by Impella before surgery, may have interference with the mitral valve due to a position change in Impella caused by manipulation for heart displacement during surgery [11].

Heart displacement in OPCAB includes heart inversion and axis torsion [12]. During heart displacement, especially heart inversion for anastomosis of the coronary arteries of the inferior wall and branchaxis torsion for anastomosis of the left circumflex, hemodynamic instability often occurs [13]. Mechanisms include reduced contraction and augmentation capacity due to heart displacement and exclusion, as well as MR during LV preload reduction and axis torsion during heart inversion [12]. Among the causes of MR, it is most likely to be exacerbated by distortion of the mitral annulus [14].

In this case, narrowing of the left intraventricular cavity caused the tip of the preoperatively implanted Impella aspirator to interfere the tendon cords of the posterior apex of the mitral valve, causing the mitral valve to become open and fixed, resulting in a poor junction with the anterior apex and more severe MR than normal heart displacement. MR decreased both systolic cardiac output and the stationary Impella volume. The diastolic stationary Impella volume was decreased by poor suction due to the intrinsic effect of the Impella suction against the LV posterior wall, resulting in an extreme drop in SBP and DBP. ECMO was added because PaO2 and rSO2 decreased regardless of respiratory support with ventilation, and SBP decreased to <90 mmHg regardless of circulatory support with Impella and catecholamine [15]. Based on body surface area calculated from height and weight, the target perfusion rate was 4.2-6.7 L/min, which could be achieved with a total Impella and ECMO flow rate of 5.0 L/min, so the Impella flow rate was reduced to 2.0 L/min [16]. Bilateral intracranial rSO2 and PaO2 in the right upper extremity then recovered to values before manipulation for heart displacement [16]. After coronary anastomosis, the patient was weaned off ECMO as PaO2 and rSO2 recovered, lactate decreased, and blood pressure rose to SBP 90 mmHg [17].

Assisted circulation by ECMO alone contributes to perfusion of organs other than the heart and oxygenation; on the other hand, retrograde perfusion from the femoral artery increases LV afterload against the heart and suppresses ejection, which requires attention [18]. However, ECMO in combination with Impella maintains general circulation and supports ejection from the left ventricle, which is useful for the treatment of cardiogenic shock [19].

For safe use of Impella during OPCAB, the patient should have a sufficient LV diameter to endure manipulation for heart displacement, while an effort should be made to control intraoperative anesthesia to prevent a reduced LV diameter due to bleeding and anesthesia [11]. Ichihara and Niinami showed good surgical results using Impella-supported CABG for patients with LVDd of 60 mm or more; therefore, this measurement may be an indicator for patient selection [11]. In addition, although the documentation states that the implantation position should be confirmed by fluoroscopy or echocardiography, confirmation not only by fluoroscopy but also by echocardiography is useful to prevent adverse events such as interference with the valve [20]. Intraoperatively, TEE is a convenient and real-time way to confirm the position of the Impella. In addition, changes in the monitoring of the arterial pressure can be monitored to detect abnormalities in the position of the Impella at an early stage.

In this case, the Impella CP was not repositioned due to the difficulty of subtly positioning the Impella CP with limited torque and the risk of falling out, which could further deteriorate consequent circulation dynamics [21].

While Impella has been reported to be useful in cases of cardiogenic shock with severely impaired cardiac function, Impella CP has also been reported to significantly increase mortality compared to IABP [1,2,22]. OPCAB has also been reported to decrease the risk of stroke and hemorrhage compared to on-pump CABG, while increasing mortality [23,24]. It is important to select the appropriate mechanical circulatory assist device for each individual patient based on preoperative cardiac function and various other risks.

After discussing the risk of adverse events associated with Impella in patients with unremarkable LV enlargement, it may be desirable to select other forms of assisted circulation, such as IABP or artificial heart-lung, but there are currently no reports demonstrating this, and further research is needed.

## Conclusions

During surgery in OPCAB, we experienced a case in which the preoperatively implanted Impella inlet approached the posterior wall of the left ventricle due to the narrowing of the LV cavity caused by heart displacement, resulting in interference with supporting tissues of the posterior mitral leaflet, resulting in severe MR and difficulty in intraoperative circulatory control.

After initiation of ECMO, perfusion pressure stabilized, and the operation was successfully completed. Sudden intraoperative conversion to artificial cardiopulmonary support should be avoided as much as possible, and the preference for mechanical circulatory assist devices such as IABP or cardiopulmonary bypass should be discussed previously in cases with unremarkable LV enlargement.
